# Supplementary material for: Bromodomain factor 5 is an essential regulator of transcription in Leishmania
Source: Nat Commun. 2022 Jul 13;13:4071. doi: 10.1038/s41467-022-31742-1 (PMC9279504; doi:10.1038/s41467-022-31742-1)
Supplement: Supplementary file 1 — Supplementary Information [file 41467_2022_31742_MOESM1_ESM.pdf]

Supplementary Information

Bromodomain factor 5 is an essential regulator of transcription in *Leishmania*

Nathaniel G. Jones[1*], Vincent Geoghegan[1], Gareth Moore[1], Juliana B. T. Carnielli[1], Katherine Newling[1], Félix Calderón[2], Raquel Gabarró[2], Julio Martín[2], Rab K. Prinjha[3], Inmaculada Rioja[3], Anthony J. Wilkinson[4], Jeremy C. Mottram[1].

1: York Biomedical Research Institute, Department of Biology, University of York, United Kingdom

2: GSK Global Health, Tres Cantos, 28760, Madrid, Spain

3: Immunology Research Unit, Research, R&D GSK, Gunnels Wood Road, Stevenage, Herts, SG1 2NY, UK

4: York Biomedical Research Institute and York Structural Biology Laboratory, Department of Chemistry, University of York, United Kingdom

Supplementary Figures

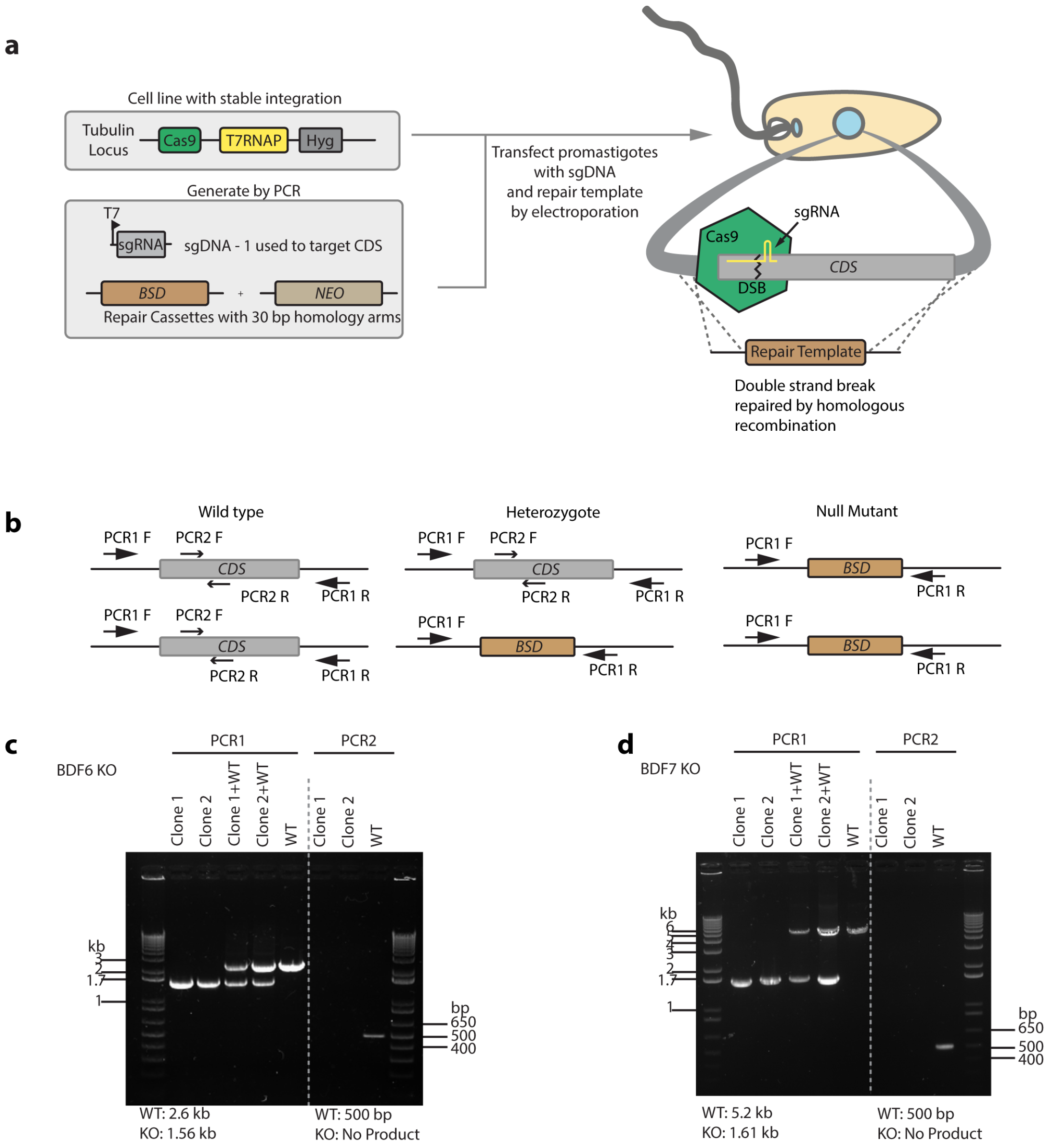

Supplementary Figure 1: CRISPR/Cas9 screening to identify non-essential bromodomain factors. a. Cartoon depicting the experimental strategy whereby *Leishmania mexicana* promastigotes constitutively expressing T7 RNA polymerase and Cas9 were transfected by electroporation with DNA to express an sgRNA targeting a BDF gene and a repair template to delete the gene of interest during the repair process. **b**. Cartoon depicting the PCR strategy to define gene knockouts isolated from CRISPR/Cas9 screening. **c**. Agarose gel PCR validation of *BDF6* null mutants. This shows two selected clones derived from separate transfections using homology flanked *BSD* cassettes. **d**. Agarose gel showing PCR validation of *BDF7* null mutants. This shows two selected clones derived from separate transfections using homology flanked *BSD* cassettes.

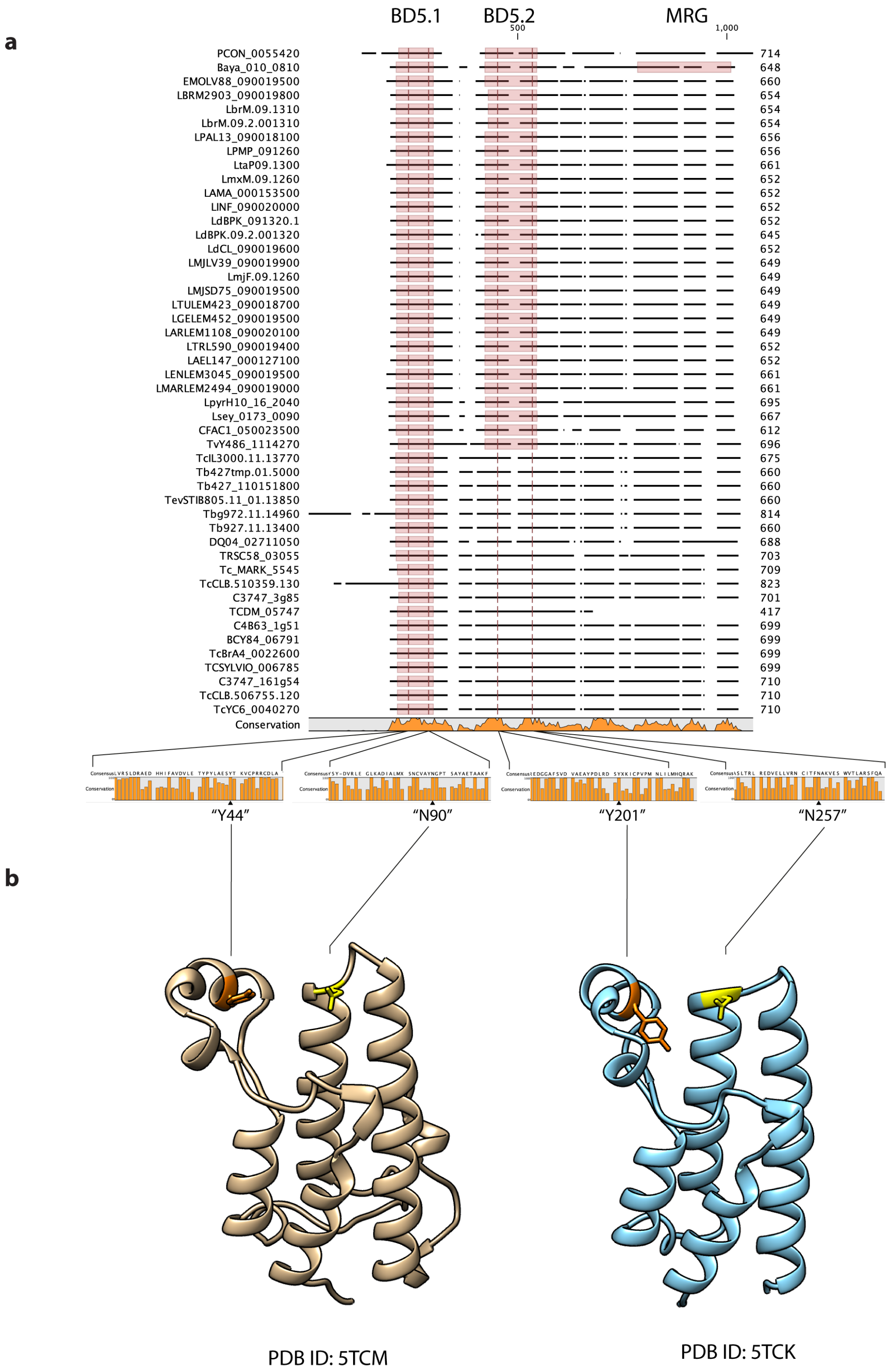

Supplementary Figure 2: CLUSTAL alignment of kinetoplastid BDF5 proteins. a. Amino acid sequences of BDF5 syntenic orthologues were aligned using the Clustal Omega plugin for CLC. Domains that were readily identifiable using the PFAM search plugin are annotated by shaded boxes, conserved tyrosine and asparagine residues are annotated by red lines within the shaded BD5.1 and BD5.2 domains. **b**. X-ray crystal structures of LdBDF5 bromodomains generated by the SGC and deposited at the PDB, conserved tyrosine residues coloured orange

and conserved asparagine residues in yellow. The notations 5TCM and 5TCK denote the PDB ID.

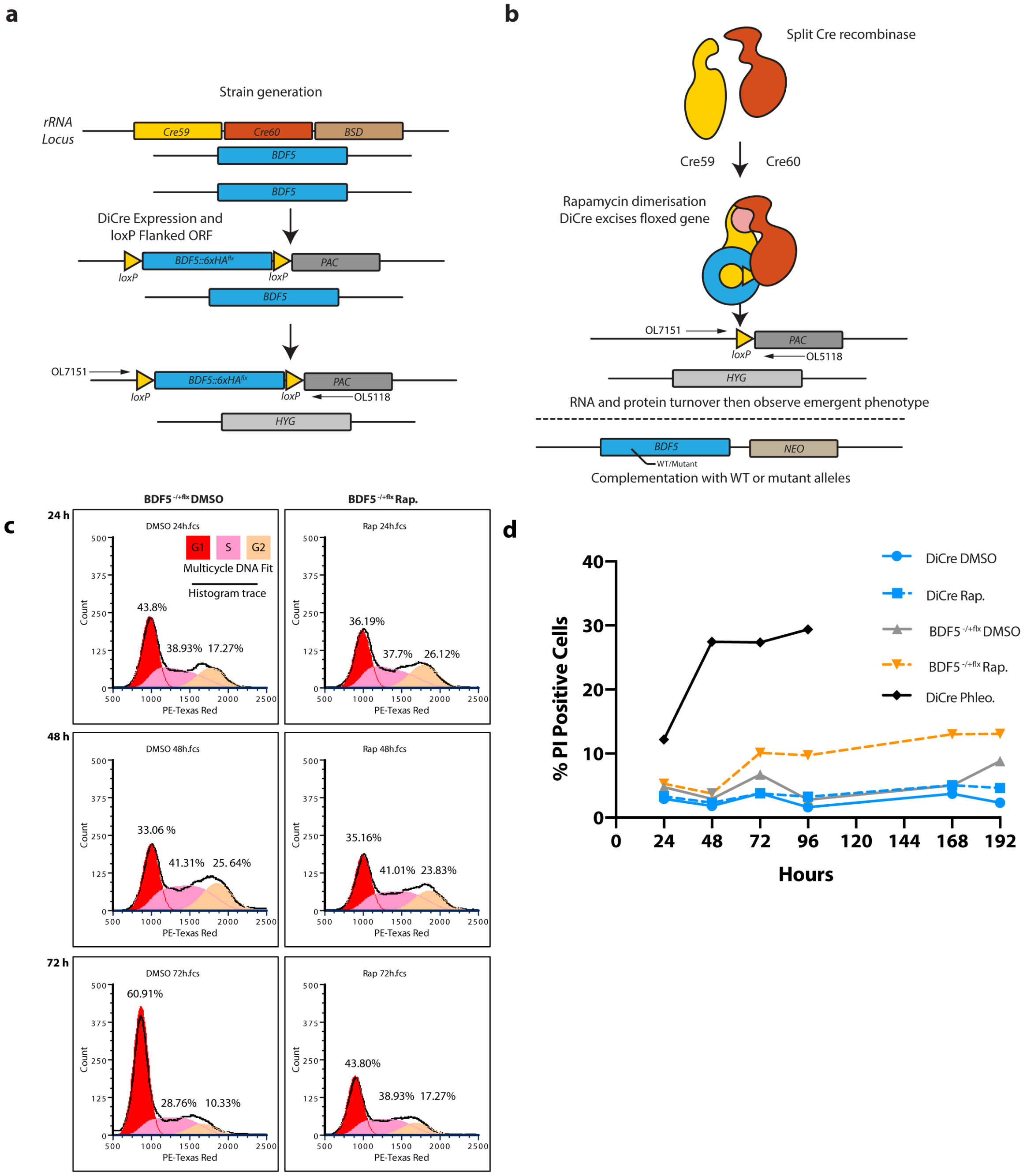

Supplementary Figure 3: Characterisation of BDF5 using DiCre Inducible gene deletion in promastigotes.

a. Cartoon representation of workflow to generate *Lmx ::DiCreΔbdf5::HYG/Δbdf5::BDF5::6xHA^{flox}*. **b**. Cartoon representation of floxed allele excision using rapamycin to dimerise the split Cre recombinase, exemplifying the ability to introduce add-back alleles for functional genetics. **c**. Flow cytometry of methanol fixed, RNAse A treated, propidium iodide stained promastigote cultures to characterise the effects of BDF5 knockout on the cell cycle over a 72 h timecourse N=20,000 events. **d**. Live/dead analysis using flow cytometry of non-fixed, propidium iodide treated promastigote cultures following BDF5

knockout. A 1 µg/ml phleomycin control was included. Points and error bars indicate mean ± standard deviation, N=20, 000 events.

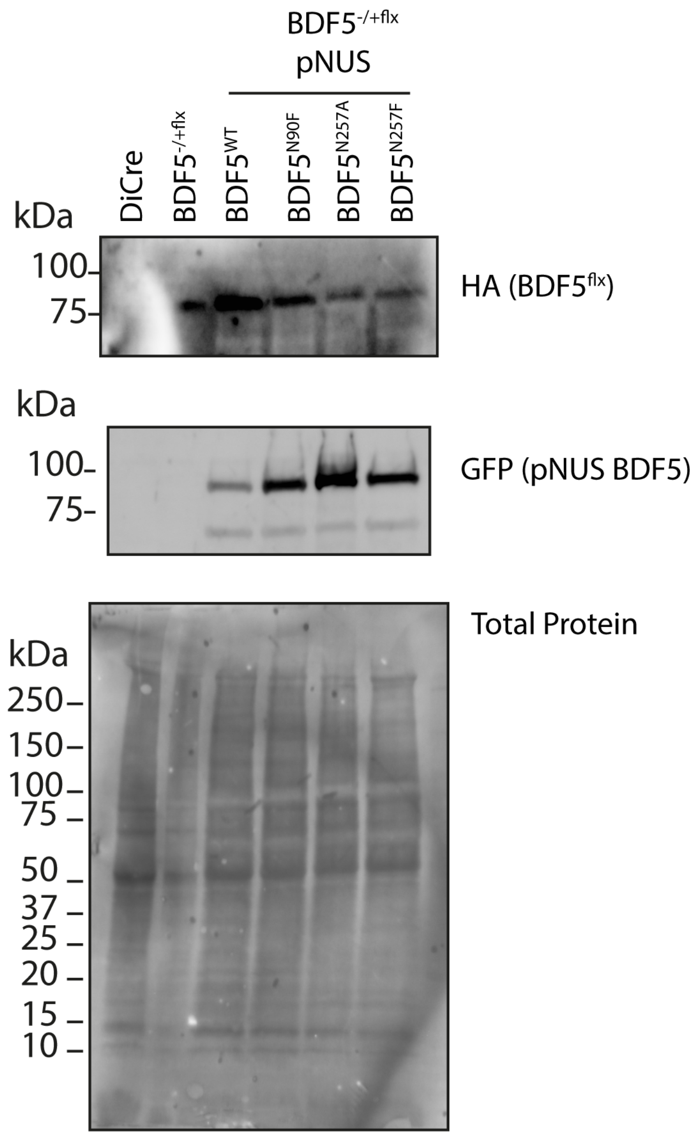

Supplementary Figure 4: Episomal Expression of BDF5 mutant alleles. Western blot analysis of cell lines expressing BDF5::GFP mutant alleles from pNUS episome vectors in the *Lmx ::DiCreΔbdf5::HYG/Δbdf5::BDF5::6xHA^{flox}*background. This blot is from a single experiment.

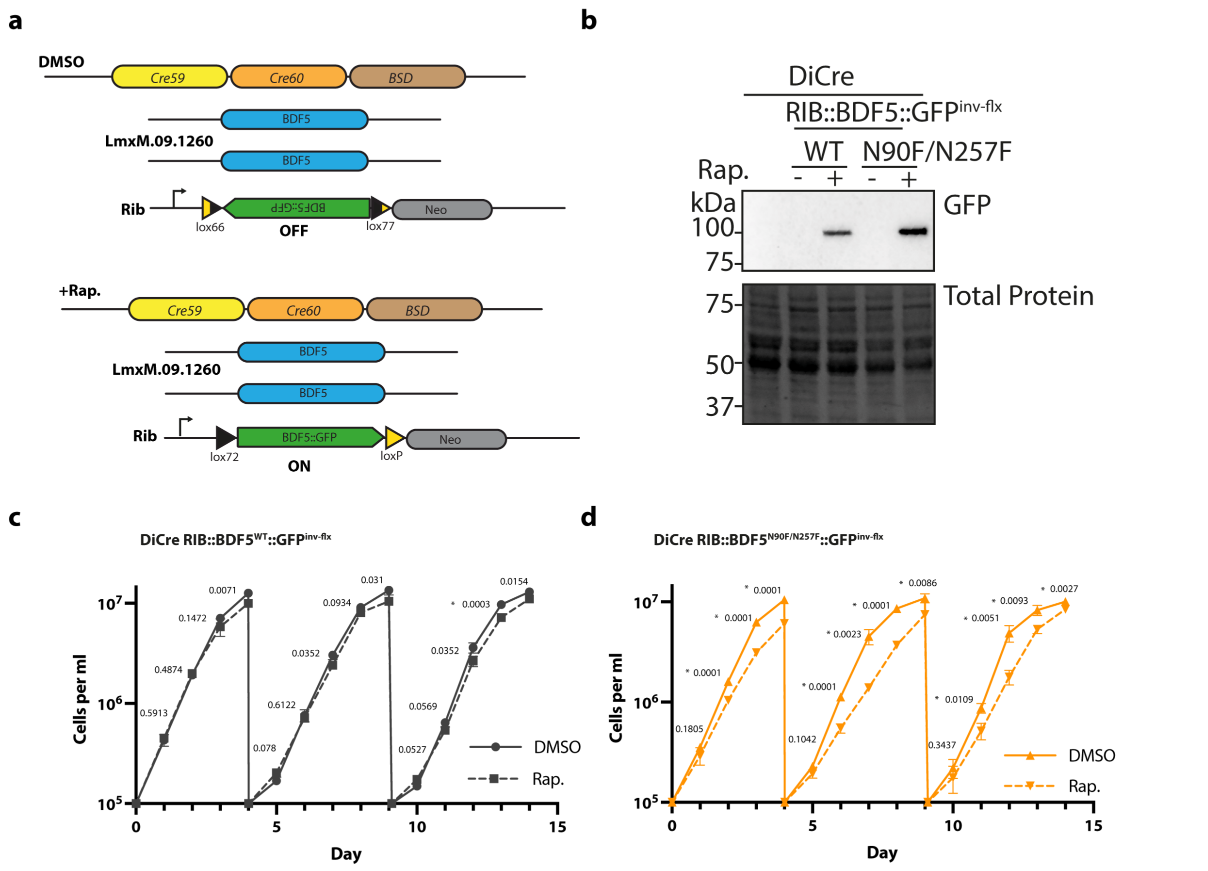

Supplementary Figure 5: Rapamycin inducible overexpression of BDF5 alleles. a. Cartoon representing the experimental set-up for expression of an extra BDF5 allele. *BDF5^N90F/N257F^::GFP* was cloned into pRIB in an inverted orientation and flanked by directional loxP sites[40] yielding *pRIB::BDF5^N90F/N257F^::GFP^inv^*. After integrating this into rRNA locus of *Lmx::DiCre,* clones were isolated and treated with 300 nM rapamycin to induce expression of the BDF5$^{N90F/N257F}$::GFP mutant protein. A control strain was also generated to express a wild-type *BDF5* allele in the same manner. **b**. Western blot analysis of cultures from C and D at the 48 h timepoint of the second growth cycle. This blot is from a single experiment. **c**. Growth curve of promastigote cultures of *DiCre::BDF5::GFP$^{inv-flx}$* treated with rapamycin or DMSO. Data points represent mean values ± standard deviation. N=3 independent culture flasks. **d**. Growth curve of promastigote cultures of *DiCre::BDF5$^{N90F/N257F}$::GFP$^{in-flxv}$* treated with rapamycin or DMSO. Data points represent mean values ± standard deviation N=3 independent culture flasks per condition. For C and D repeated two-sided t-tests were performed using Prism (GraphPad) corrected with Benjamini and Hochberg method, p-values are annotated and those marked * denote significant discoveries.

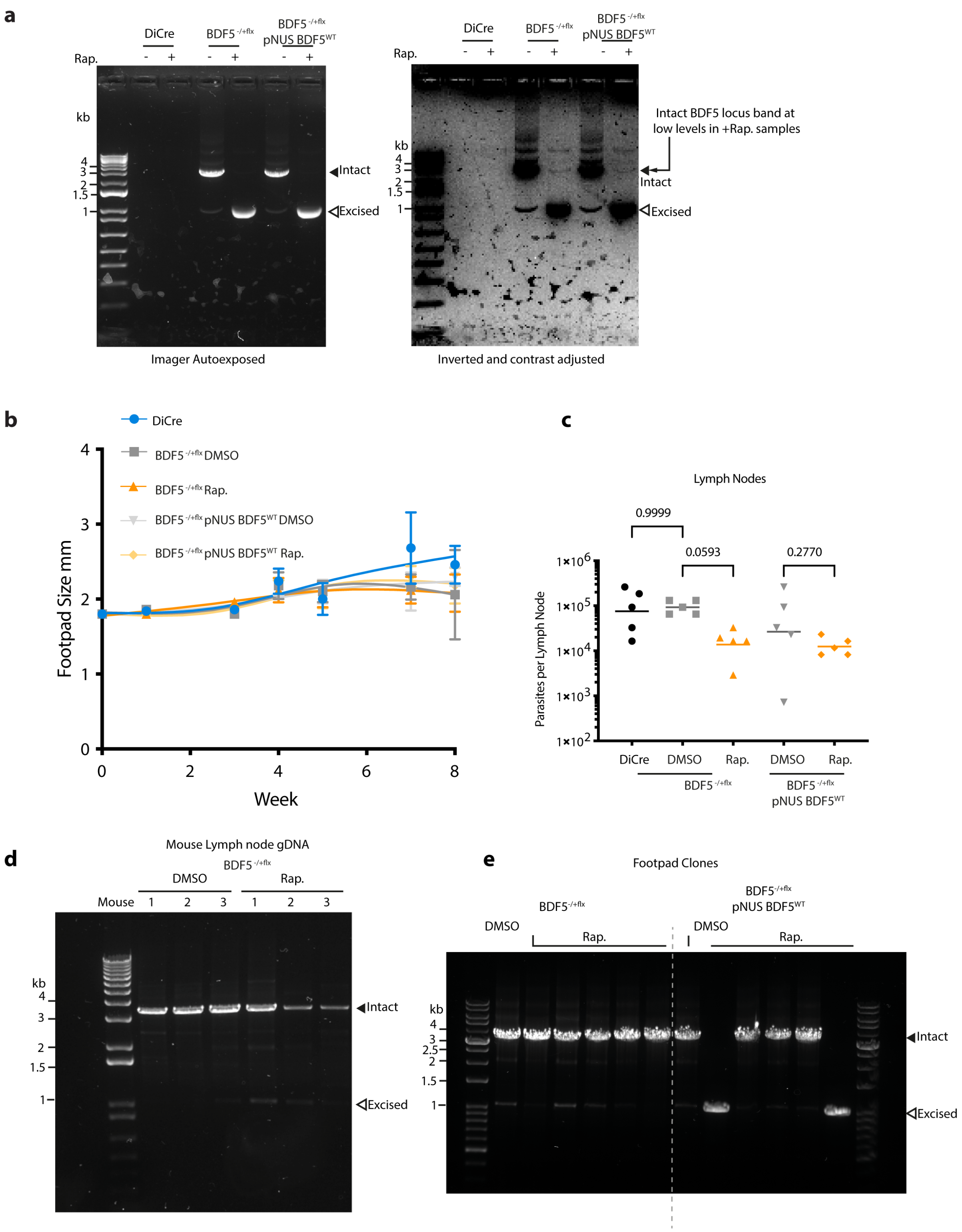

Supplementary Figure 6: Characterisation of BDF5 using DiCre inducible gene deletion in murine infection. a. PCR and agarose gel analysis of stationary phase cultures used to infect mice. This blot is from a single experiment. **b.** Measurements of footpad size of mice infected with indicated parasite strains. Points and error bars indicate mean ± standard deviation, N=5. **c.** Parasite burdens from infected mouse popliteal lymph nodes determined by limiting dilution, individual points for each mouse with median values indicated by line. Comparisons of Kruskal-Wallace test with Dunn's correction indicate by lines, associated p-values written

above, n=5. **d**. Agarose gel exemplifying PCR analysis of genomic DNA extracted from popliteal lymph nodes of mice infected with the BDF5::6xHA$^{-/+flx}$ cultures that were treated with rapamycin or not, indicating retention of the BDF5 allele at 8-weeks post-infection. **e.** PCR and agarose gel analysis exemplifying clones surviving as promastigotes following clonogenic assay to detect *BDF5*$^{-/+flx}$ allele. These gels are from the single mouse experiment.

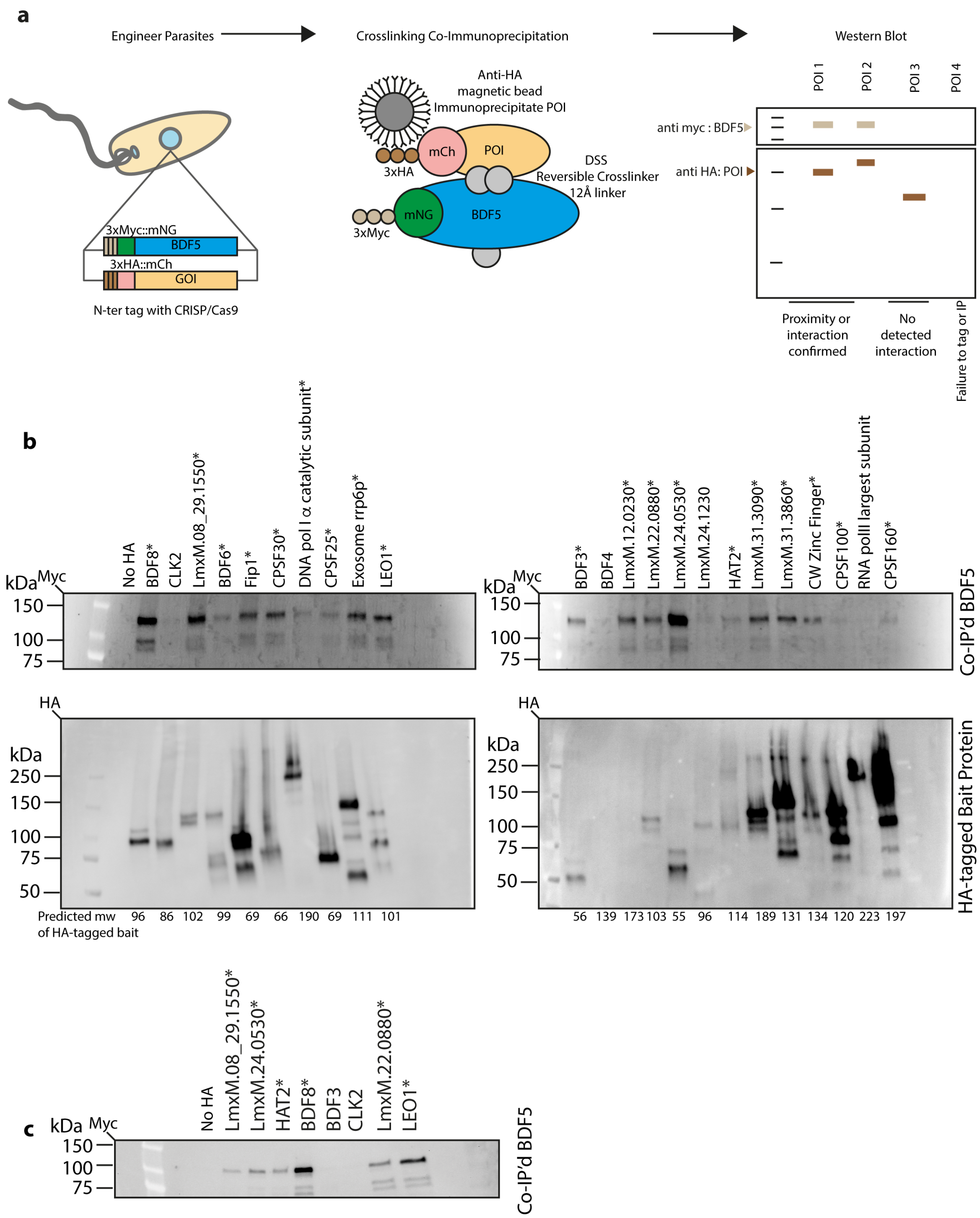

Supplementary Figure 7: Co-immunopreciptation analysis of BDF5-proximal proteins. a. cartoon of experimental workflow. BDF5-proximal proteins identified by XL-BioID proteins were HA-tagged in the *LmxT7/Cas9 3xMyc::mNG::BDF5* strain to generate a panel of cell lines

containing both a HA-tagged protein of interest (POI) and Myc-tagged BDF5. **b**. The HA-tagged proteins were used as bait in a crosslinking, anti-HA immunoprecipitation and co-precipitating (co-IP). Myc-tagged BDF5 protein is detected using western blot with anti-Myc (Upper Panel). Precipitation of the bait protein was confirmed by western blot (lower panel). The presence of BDF5 co-precipitating with a bait POI confirms the XL-BioID observations as being robust. These blots represent a single experiment. **c**. A selection of HA-tagged proteins likely to be CRKT complex members were immunoprecipitated without prior crosslinking and the elution fractions probed for co-precipitating BDF5. A BDF5 positive band here suggest the interaction of the bait protein with BDF5 is relatively stable. This blot represents a single experiment.

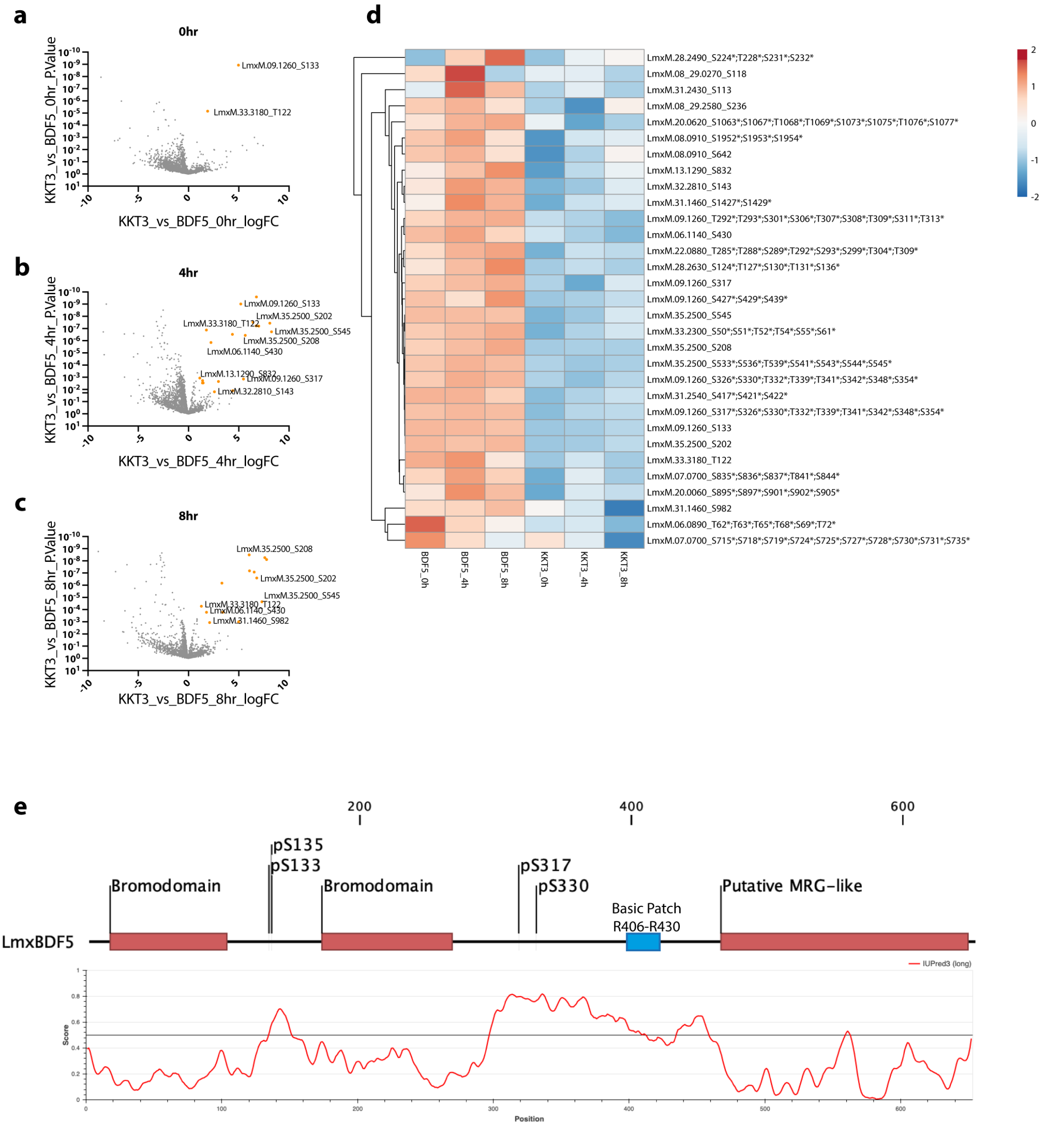

Supplementary Figure 8: Proximity phosphoproteomic analysis of BDF5 across the cell cycle. a, b and **c**. Volcano plots of phosphopeptide enrichment and confidence over 0 h, 4 h and 8 h release from hydroxyurea synchronisation. Ambiguous phosphosite localisations are denoted with a *. The log2 fold change and p-values are derived from limma analysis using comparing against KKT3-miniTurbo and using the eBayes function. Multiple testing correction was carried out according to Benjamini & Hochberg, false discovery rate for proximal phosphosites was 5%. **d**. Heatmap of proximal phosphosites represented by median values of 5 replicates after $\log_2$+1 transformation and data centring. Samples depicted are the BDF5 and KKT3 0 h, 4 h and 8 h timepoints. **e**. Architecture of BDF5 protein depicting the locations of identified phosphorylation sites. The lower panel depicts regions predicted to be intrinsically disordered using IUPred3, indicating the detected phosphosites occur in regions of BDF5 likely to be unstructured.

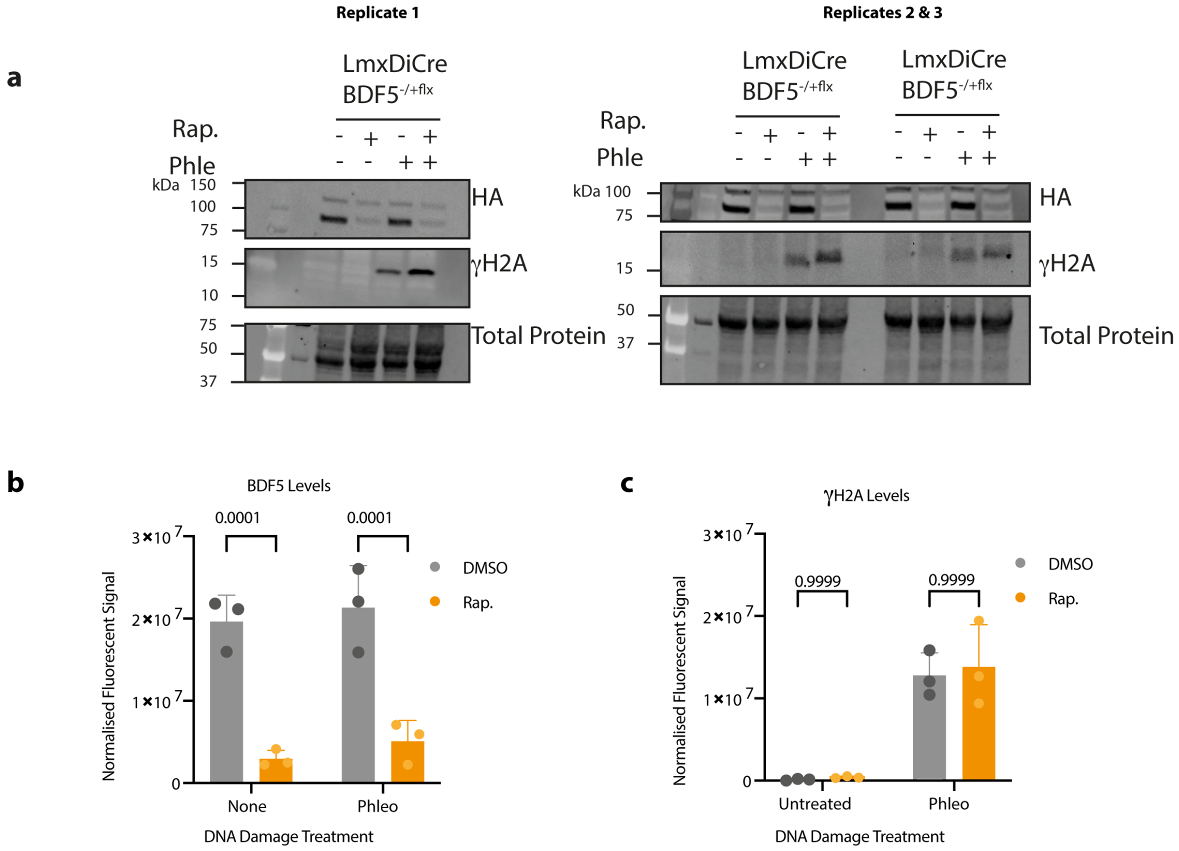

Supplementary Figure 9: DNA damage response in BDF5 depleted cells. a. Western blot analysis of cultures treated with DMSO or Rapamycin to delete the floxed allele of *BDF5* in *BDF5*$^{-/+flx}$ for 48 h followed by passaging to 2 x 10^5 cells **ml**$^{-1}$, another 48 h of rapamycin treatment with the final 24 h in the presence or absence of 1 µg **ml**$^{-1}$ of phleomycin (Phle). BDF5::6xHA levels and γH2A levels were assessed by Western blot with anti-HA and anti- γH2A antibodies respectively. (N=3). **b**. Normalised quantification of chemiluminescent signal of BDF5 bands. Data points and bars indicate mean ± SD, N=3. **c**. Normalised quantification of chemiluminescent signal of γH2A bands. Data points and bars indicate mean± SD, data compared by 2-way ANOVA, p-values indicated above. N=3 independent experiments.

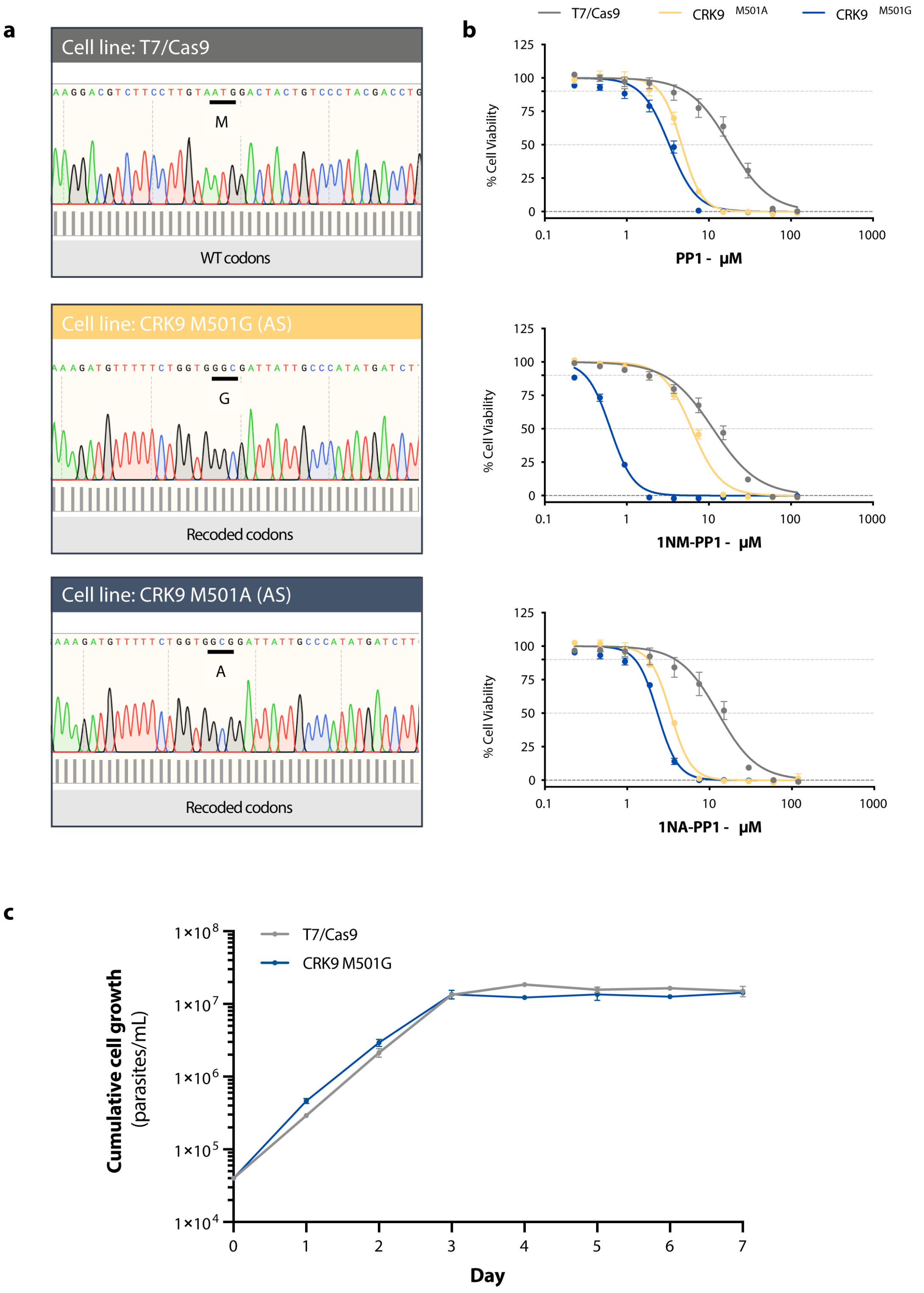

Supplementary Figure 10: Generation of CRK9 analog-sensitised strains. a. Sanger sequencing of *CRK9* in *L. mexicana T7/Cas9* and precision-edited mutants showing homozygous strains encoding small gatekeeper mutations M501G and M501A. **b**. Dose-response curves of promastigote cell viability after 72 h treatment in varying concentrations of bulky-kinase inhibitors PP1, 1NM-PP1 and 1NA-PP1, measured by Alamar blue method, mean±SD, n=3. **c**. Growth curve of promastigote cultures of T7/Cas9 and the CRK9^{M501A} strain indicating the small

gatekeeper residue does not impact growth of promastigotes, mean±SD, N=3 culture flasks per condition.

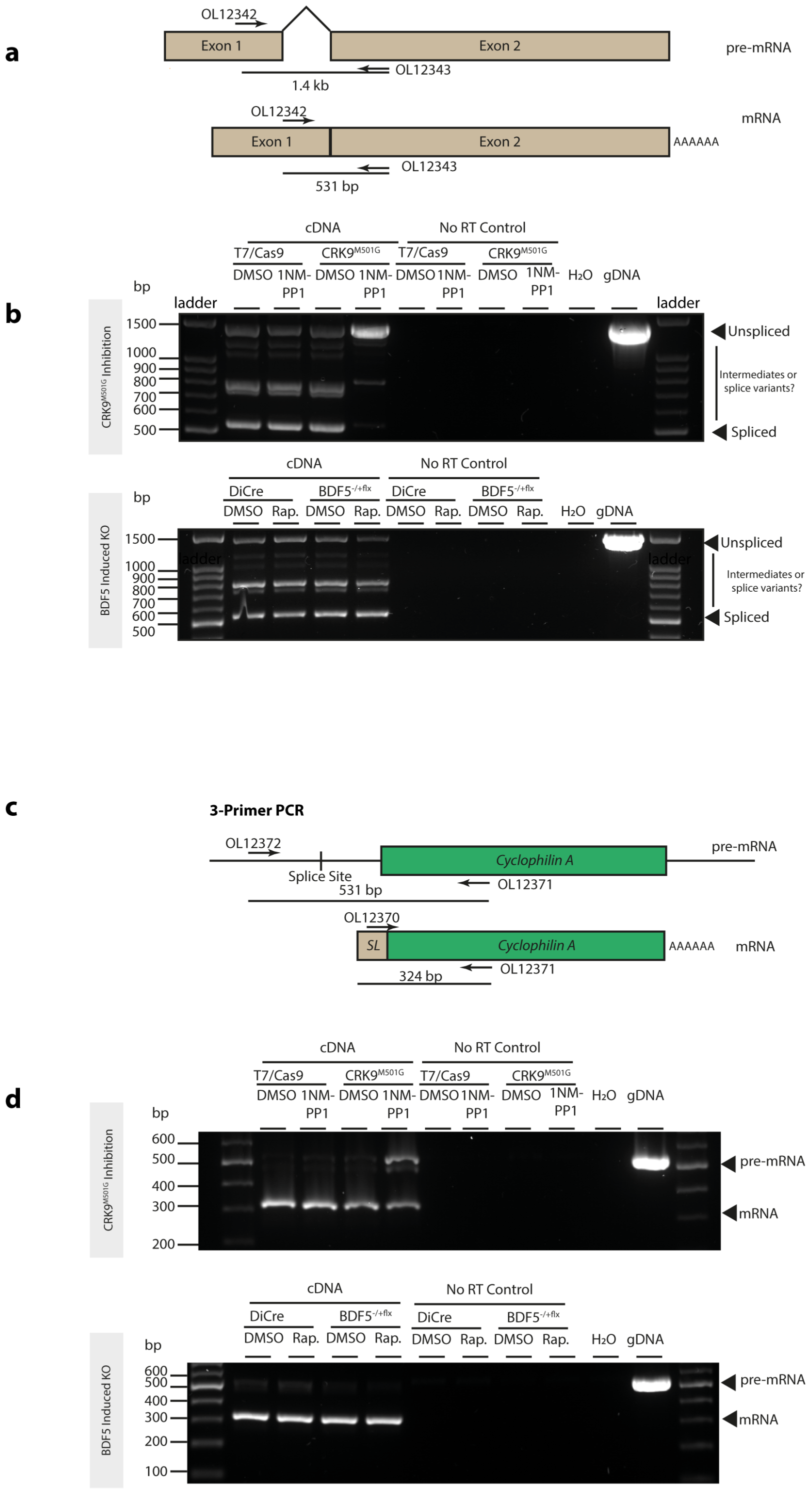

Supplementary Figure 11: Effect of BDF5 on cis- and trans-splicing of mRNA. a. Cartoon showing the strategy of the RT-PCR assay to detect cis-splicing of polyA-polymerase mRNA (LmxM.08_29.2600) after CRK9 inhibition (a positive control for splicing defects) or BDF5 deletion. Cells were treated with DMSO, Rapamycin or 1NM-PP1. **b**. Agarose gel of RT-PCR assay to detect cis-splicing of polyA-polymerase mRNA (LmxM.08_29.2600) after CRK9

inhibition or BDF5 deletion. cDNA prepared using random hexamers was used to prime the assay, thus capturing the pre-mRNA and mRNA. *L. mexicana* T7/Cas9 was used as the control strain for the CRK9 analog-sensitised strain. Accumulation of the pre-mRNA is only observed when $CRK9^{AS}$ is inhibited with 1NM-PP1. No-RT controls were included to exclude gDNA contamination. H_2O indicates a water control to exclude master mix contamination, and gDNA was used as a positive control to exemplify the unspliced band size. This gel represents a single experiment, this experiment was conducted 3 times with the same result. **c**. Cartoon showing the strategy of the triple-primer RT-PCR assay to detect trans-splicing of Cyclophilin A mRNA (LmxM.25.0910) after CRK9 inhibition or BDF5 deletion. **d**. Agarose gel of triple-primer RT-PCR assay to detect trans-splicing of Cyclophilin A mRNA (LmxM.25.0910) after CRK9 inhibition or BDF5 deletion. cDNA prepared using random hexamers was used to prime the assay, thus capturing the pre-mRNA and mRNA. No-RT controls were included to exclude gDNA contamination. H_2O indicates a water control to exclude master mix contamination, and gDNA was used as a positive control to exemplify the unspliced band size. Accumulation of the pre-mRNA is only observed when $CRK9^{AS}$ is inhibited with 1NM-PP1. This gel represents a single experiment.

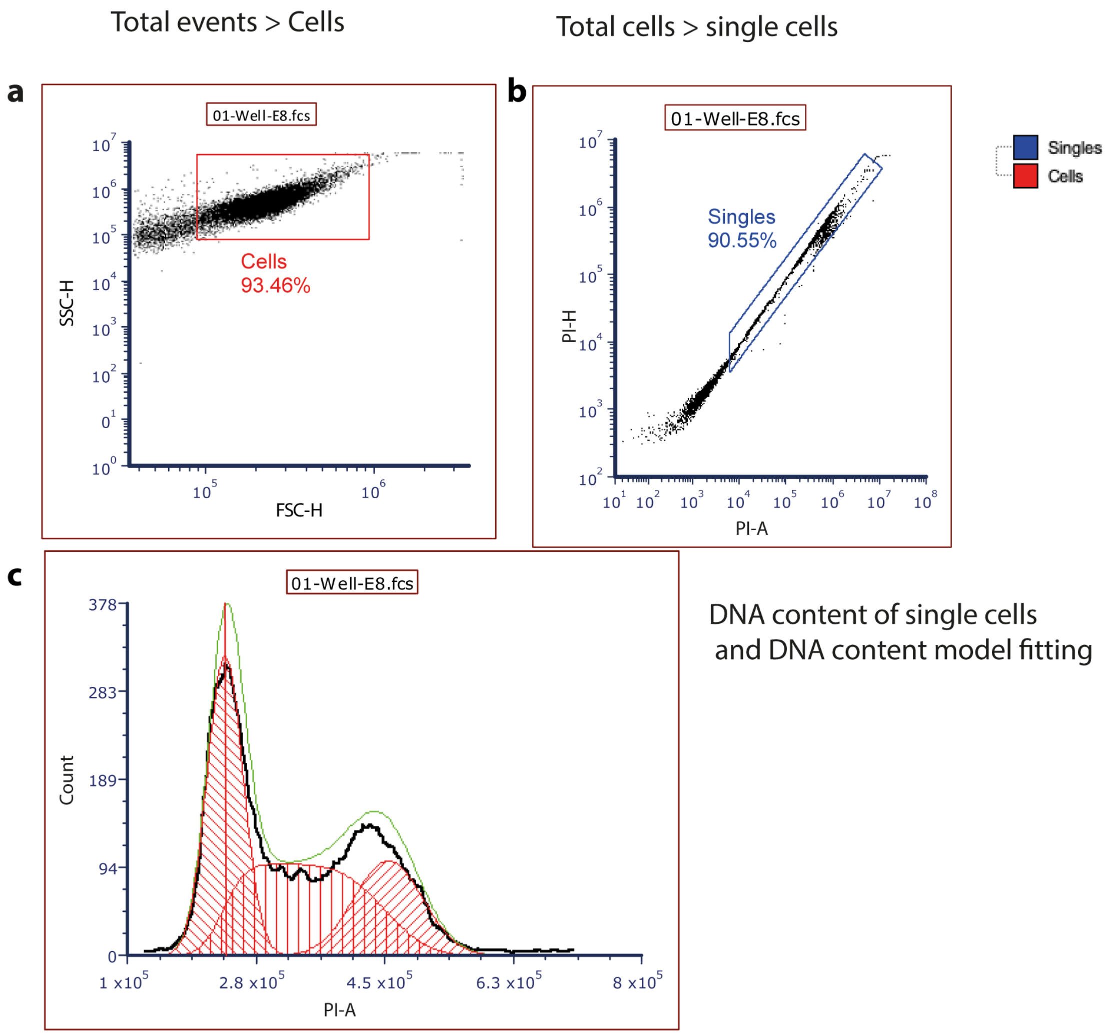

Cycle	G1 Mean	G1 CV	%G1	G2 Mean	G2 CV	%G2	%S	G2/G1	%Total	B.A.D
Diploid	233155.52	10.66	37.00	456317.51	10.31	22.28	40.72	1.97	100.00	0.00

Supplementary Figure 12: Flow cytometry gating strategy. a. An example of the gating strategy used for cell cycle analysis. Cells were analysed using a Beckman Coulter Cyan ADP flow cytometer. Data was analysed using FCS Express 7 Flow version 7.10.0007 (De Novo Software, Inc.). **a**. Cells were acquired based on FSC-H and SSC-H. **b.** Singlets were gated by comparing propidium iodide signal PI-area (PI-A) to PI-height (PI-H). **c.** Cell-cycle state was determined using PI-A from the histogram using the Multicycle DNA module (model 6) for cell-cycle analysis.
