# Supplementary material for: Bromodomain factor 5 is an essential regulator of transcription in Leishmania
Source: Nat Commun. 2022 Jul 13;13:4071. doi: 10.1038/s41467-022-31742-1 (PMC9279504; doi:10.1038/s41467-022-31742-1)
Supplement: Supplementary file 3 — Description of additional supplementary files [file 41467_2022_31742_MOESM3_ESM.pdf]

Description of Additional Supplementary Files

Supplementary Data 1: Excel spreadsheet, List of Oligonucleotides, plasmids, cell lines and antibodies used in this study.

Supplementary Data 2: Excel spreadsheet, SAINTq Analysis of BDF5 XL-BioID data to identify proximal proteins, also contains list of remote homology identified in hypothetical proteins by HHPRED analysis.

Supplementary Data 3: Excel spreadsheet, BDF5 proximal phosphosites through the cell cycle as determined by limma analysis of phosphoproteomic XL-BioID samples.

Supplementary Software: Custom Python code to call peaks once wig files have been obtained from deepTools.
