# Supplementary material for: Bromodomain factor 5 is an essential regulator of transcription in Leishmania
Source: Nat Commun. 2022 Jul 13;13:4071. doi: 10.1038/s41467-022-31742-1 (PMC9279504; doi:10.1038/s41467-022-31742-1)
Supplement: Supplementary file 7 — Python Code for ChIP peak calling [file 41467_2022_31742_MOESM7_ESM.zip › Python Code for ChIP-seq analysis.docx]

**Code Availability**

**Bromodomain factor 5 is an essential regulator of transcription in *Leishmania***

Nathaniel G. Jones^1*^, Vincent Geoghegan^1^, Gareth Moore^1^, Juliana B. T. Carnielli^1^, Katherine Newling^1^, Félix Calderón^2^, Raquel Gabarró^2^, Julio Martín^2^, Rab K. Prinjha^3^, Inmaculada Rioja^3^, Anthony J. Wilkinson^4^, Jeremy C. Mottram^1^.

**Custom Python code to call peaks once wig files have been obtained from deepTools.**

# combine wigs by taking mean with python script:

(base) Katherines-MacBook-Pro:20220419_control_HA_chipseq kn675$ cat combine_wigs.py

import numpy as np

log2ratio_dict = {}

wig1 = open("log2ratio_1_normalised_SES_bin500_control.wig")

wig2 = open("log2ratio_2_normalised_SES_bin500_control.wig")

for line in wig1:

if not line.startswith('#'):

v1,v2,v3,log2ratio = line.split()

key = v1+':'+v2+':'+v3

if key not in log2ratio_dict:

log2ratio_dict[key]={}

log2ratio_dict[key]['wig1'] = float(log2ratio)

for line in wig2:

if not line.startswith('#'):

v1,v2,v3,log2ratio = line.split()

key = v1+':'+v2+':'+v3

if key not in log2ratio_dict:

log2ratio_dict[key]={}

log2ratio_dict[key]['wig2'] = float(log2ratio)

outfile = open("log2ratio_all_normalised_SES_bin500_mean_control.wig", 'w')

for key in log2ratio_dict:

if 'wig1' in log2ratio_dict[key] and 'wig2' in log2ratio_dict[key]:

meanratio = np.mean([float(log2ratio_dict[key]['wig1']),float(log2ratio_dict[key]['wig2'])])

chrom,start,end = key.split(':')

outfile.write(chrom+'\t'+start+'\t'+end+'\t'+str(meanratio)+'\n')

(base) Katherines-MacBook-Pro:20220419_control_HA_chipseq kn675$ python combine_wigs.py

# filtered the file for peaks by only including bins with value >0.5

(base) Katherines-MacBook-Pro:20220419_control_HA_chipseq kn675$ cat filter_wig.py

wig1 = open("log2ratio_all_normalised_SES_bin500_mean_control.wig")

wig1_filtered = open("log2ratio_all_normalised_SES_bin500_mean_thresh05_control.wig", 'w')

saved_lines = []

for line in wig1:

if line.startswith('#'):

wig1_filtered.write(line)

elif float(line.split()[3])>0.5:

if len(saved_lines)>1:

for l in saved_lines:

wig1_filtered.write(l)

wig1_filtered.write(line)

saved_lines = []

else:

saved_lines.append(line)

else:

saved_lines = []

(base) Katherines-MacBook-Pro:20220419_control_HA_chipseq kn675$ python filter_wig.py

# merge peaks that are less than 5kb from each other

(base) Katherines-MacBook-Pro:20220419_control_HA_chipseq kn675$ cat merge_wig_peaks.py

wig = open("log2ratio_all_normalised_SES_bin500_mean_thresh05_control.wig")

peak_file = open("final_merged_peaks_05_control.wig", 'w')

prev_chrom,prev_start,prev_end,prev_log2ratio = ['',0,0,0]

peaks = []

peak_start = 0

peak_end = 0

for line in wig:

chrom,start,end,log2ratio = line.split()

if chrom!=prev_chrom:

if chrom!='LmxM.01':

peaks.append(prev_chrom+':'+str(peak_start)+':'+str(peak_end))

peak_file.write(prev_chrom+'\t'+str(peak_start)+'\t'+str(peak_end)+'\t'+'1'+'\n')

# add new peak at end of previous chrom

peak_start = start

peak_end = end

elif chrom==prev_chrom and int(start)==int(prev_start)+500:

# peak_start stays same

peak_end = end

elif chrom==prev_chrom and int(start)-5000<int(peak_end):

# peak_start stays same

print("peak merge")

peak_end = end

elif chrom==prev_chrom and int(start)-5000>int(peak_end):

# add new peak

peaks.append(chrom+':'+str(peak_start)+':'+str(peak_end))

peak_file.write(chrom+'\t'+str(peak_start)+'\t'+str(peak_end)+'\t'+'1'+'\n')

# reset peak to new bin

peak_start = int(start)

peak_end = int(end)

prev_chrom,prev_start,prev_end,prev_log2ratio = line.split()

print(peaks)

(base) Katherines-MacBook-Pro:20220419_control_HA_chipseq kn675$ python merge_wig_peaks.py
