# Supplementary material for: Bromodomain factor 5 is an essential regulator of transcription in Leishmania
Source: Nat Commun. 2022 Jul 13;13:4071. doi: 10.1038/s41467-022-31742-1 (PMC9279504; doi:10.1038/s41467-022-31742-1)
Supplement: Supplementary file 8 — Reporting Summary [file 41467_2022_31742_MOESM8_ESM.pdf]

nature portfolio

Corresponding author(s): Nathaniel G Jones

Last updated by author(s): Jun 16, 2022

Reporting Summary

Nature Portfolio wishes to improve the reproducibility of the work that we publish. This form provides structure for consistency and transparency in reporting. For further information on Nature Portfolio policies, see our Editorial Policies and the Editorial Policy Checklist.

Statistics

For all statistical analyses, confirm that the following items are present in the figure legend, table legend, main text, or Methods section.

n/a	Confirmed	
☐	☒	The exact sample size (*n*) for each experimental group/condition, given as a discrete number and unit of measurement
☐	☒	A statement on whether measurements were taken from distinct samples or whether the same sample was measured repeatedly
☐	☒	The statistical test(s) used AND whether they are one- or two-sided<br>*Only common tests should be described solely by name; describe more complex techniques in the Methods section.*
☐	☒	A description of all covariates tested
☐	☒	A description of any assumptions or corrections, such as tests of normality and adjustment for multiple comparisons
☐	☒	A full description of the statistical parameters including central tendency (e.g. means) or other basic estimates (e.g. regression coefficient) AND variation (e.g. standard deviation) or associated estimates of uncertainty (e.g. confidence intervals)
☐	☒	For null hypothesis testing, the test statistic (e.g. *F*, *t*, *r*) with confidence intervals, effect sizes, degrees of freedom and *P* value noted<br>*Give P values as exact values whenever suitable.*
☒	☐	For Bayesian analysis, information on the choice of priors and Markov chain Monte Carlo settings
☒	☐	For hierarchical and complex designs, identification of the appropriate level for tests and full reporting of outcomes
☐	☒	Estimates of effect sizes (e.g. Cohen's *d*, Pearson's *r*), indicating how they were calculated

Our web collection on statistics for biologists contains articles on many of the points above.

Software and code

Policy information about availability of computer code

Data collection	Summit 4.2 Beckman Coulter<br>Zeiss Zen Blue (V2.3.69.1005)<br>Xcalibur software (version 4.0, Thermo)
Data analysis	Graphpad Prism Version 9<br>Ziess Zen Blue (V2.3.69.1005)<br>Fiji Version 2.0.0<br>ImageLab 6.0.0<br>Progenesis QI (Version 2.2., Waters)<br>Mascot Daemon (version 2.6.1, Matrix Science)<br>SAINTq v0.0.4 (PMID: 27119218)<br>Circa (Version 1.2.1 (1.2.1)) (OMGenomics)<br>FCS Express 7 Flow version 7.10.0007 (De Novo Software, Inc.)<br><br>FastQC version 11.0.5 and Cutadapt version 2.5, respectively. This was followed by alignment to the L. mexicana T7/Cas9 genome using BWA-MEM (version 0.7.17). Paired ChIP-seq and input alignment files were normalised to each other using deepTools' bamCompare (version 3.3.1)

For manuscripts utilizing custom algorithms or software that are central to the research but not yet described in published literature, software must be made available to editors and reviewers. We strongly encourage code deposition in a community repository (e.g. GitHub). See the Nature Portfolio guidelines for submitting code & software for further information.

Data

Policy information about availability of data

All manuscripts must include a data availability statement. This statement should provide the following information, where applicable:

- Accession codes, unique identifiers, or web links for publicly available datasets
- A description of any restrictions on data availability
- For clinical datasets or third party data, please ensure that the statement adheres to our policy

Mass spectrometry data sets and proteomic identifications are available to download from MassIVE (MSV000087750), [doi:10.25345/C5G543] and ProteomeXchange (PXD027080).
ChIP-Seq and RNA-Seq reads are available as FASTQ files at the European Nucleotide Archive under the accession code PRJEB46800. Uncropped versions of any cropped western blots and PCR gels are provided with this paper as a separate PDF.

Genome FASTA files and GFF files were retrieved from TriTrypDB (https://tritrypdb.org/tritrypdb/app).
Structures of Leishmania donovani BDF5 bromodomains were retrieved from the Protein Data Bank (https://www.rcsb.org/) using the PDB IDs 5TCM and 5TCK.

Field-specific reporting

Please select the one below that is the best fit for your research. If you are not sure, read the appropriate sections before making your selection.

☒ Life sciences ☐ Behavioural & social sciences ☐ Ecological, evolutionary & environmental sciences

For a reference copy of the document with all sections, see nature.com/documents/nr-reporting-summary-flat.pdf

Life sciences study design

All studies must disclose on these points even when the disclosure is negative.

Sample size	Sample size for mouse infections were chosen based on prior experience with deleting essential genes in a DiCre strain, PMID: 32544189. With effect sizes and standard deviations of the DUB2 mutant, experiments using groups of 5 mice give a power factor of 0.8 assuming a 5% type I error rate.
Data exclusions	Data were excluded from some of the RNA seq samples based on the following criteria: Genes with <10 reads mapping were excluded from being graphed on figure 6B. No such exclusions were made for the metaplot anylses in the following figures, 6C onwards.
Replication	Experimental replicates are specified in the figure legends. All attempts at replication were successful.<br>ChIP-seq of BDF5 was performed in triplicate, for the control strain it was performed in duplicate. RNA seq was carried out in triplicate.
Randomization	Cell biology: Blinding was not possible for the cell/molecular biology experiments, but where possible we used spike-in controls as normalisation channels/internal controls. Vehicle treatment (DMSO) of cultures was always performed when comparing to rapamycin treated samples. Control and test flasks were cultured under the same controlled conditions. Loading controls of total protein were always presented for western blots with the exception of the immunoprecipitations, due to the variable amount and composition of material retrieved, however, untagged, and parental controls were included in these experiments.<br><br>Animal studies: animals were selected randomly for each group.
Blinding	Animal studies: Blinding was not possible in the animal models.

Reporting for specific materials, systems and methods

We require information from authors about some types of materials, experimental systems and methods used in many studies. Here, indicate whether each material, system or method listed is relevant to your study. If you are not sure if a list item applies to your research, read the appropriate section before selecting a response.

Materials & experimental systems

n/a	Involved in the study	
☐	☒	Antibodies
☐	☒	Eukaryotic cell lines
☒	☐	Palaeontology and archaeology
☐	☒	Animals and other organisms
☒	☐	Human research participants
☒	☐	Clinical data
☒	☐	Dual use research of concern

Methods

n/a	Involved in the study	
☐	☒	ChIP-seq
☐	☒	Flow cytometry
☒	☐	MRI-based neuroimaging

Antibodies

Antibodies used	All antibodies used are defined in Table S1
Validation	Antibodies against epitope tags were validated by including lysate from unmodified leishmania strains on any western blot, to ensure there were no non-specific, crossreaction bands to parasite proteins that were not tagged with the relevant epitope. Molecular weight of bands were checked against predicted expected sizes of the tagged proteins.<br><br>Individual antibodies were validated by manufacturers by western blotting against epitope tagged fusion proteins. Details are found at the following websites<br>anti-Myc Affinity purified polyclonal Chicken A190-103A https://www.thermofisher.com/antibody/product/Myc-Tag-Antibody-Polyclonal/A190-103A<br>anti-HA 16B12 Mouse 901501 https://www.biolegend.com/fr-ch/products/purified-anti-ha-11-epitope-tag-antibody-11374<br>anti-HA Affinity purified polyclonal Rabbit A190-108A https://www.thermofisher.com/antibody/product/HA-Tag-Antibody-Polyclonal/A190-108A<br>anti-HA Beads Monoclonal Mouse 88836 https://www.thermofisher.com/order/catalog/product/88836<br>anti-GFP JL-8 Mouse 632380 https://www.takarabio.com/products/antibodies-and-elisa/fluorescent-protein-antibodies/green-fluorescent-protein-antibodies?catalog=632380<br><br>anti-gamma-H2A Polyclonal Rabbit was validated by David Horn's lab in the following paper. PMID: 22353557 We validated this against Leishmania mexicana by performing western blots against untreated cells, and cells treated with the DNA damaging agent , Phleomycin. A single band of the expected ~15-20 kDa size appeared only in the phleomycin treated cells, consistent with a cellular response to DNA damage.

Eukaryotic cell lines

Policy information about cell lines

Cell line source(s)	Leishmania mexicana (MNYC/BZ/62/M379) expressing T7 RNA Pol and SpCas9 was published in Beneke et al (2017) R Soc Open Sci 4:170095
Authentication	Cell lines derived in this study were verified by PCR , or in the case of LmxDiCreBDF5-/+flx this line was whole genome sequenced as part of our chipseq workflow. Epitope tagged strains were verified by western blotting.
Mycoplasma contamination	Leishmania strains were not tested for mycoplasma.
Commonly misidentified lines (See ICLAC register)	No commonly misidentified cell lines were used in this study.

Animals and other organisms

Policy information about studies involving animals; ARRIVE guidelines recommended for reporting animal research

Laboratory animals	BALB/c mice, female, 4-6 weeks. All mice were housed in IVC caging and ambient temperatures are between 19-23C, humidity levels were 55% plus or minus 10%. There was a 12 hour light/dark cycle, 7.30am to 7.30pm.
Wild animals	No wild animals were used in this study.
Field-collected samples	Field collected samples were not used in this study.
Ethics oversight	All experiments were conducted according to the Animals (Scientific Procedures) Act of 1986, United Kingdom, and had approval from the University of York Animal Welfare and Ethical Review Body (AWERB) committee. All animal studies were ethically reviewed and carried out in accordance with Animals (Scientific Procedures) Act 1986 and the GSK Policy on the Care, Welfare and Treatment of Animals.

Note that full information on the approval of the study protocol must also be provided in the manuscript.

ChIP-seq

Data deposition

☒ Confirm that both raw and final processed data have been deposited in a public database such as GEO.

☒ Confirm that you have deposited or provided access to graph files (e.g. BED files) for the called peaks.

Data access links *May remain private before publication.*	ChIP-Seq and RNA-Seq reads are available as FASTQ files at the European Nucleotide Archive under the accession code PRJEB46800. Processed data being deposited at TriTrypDB https://tritrypdb.org/tritrypdb/app/
Files in database submission	SAMEA9541422 - BDF5 Elution Replicate 1<br>SAMEA9541423 - BDF5 Elution Replicate 2<br>SAMEA9541424 - BDF5 Elution Replicate 3

SAMEA9541425 - BDF5 Input Replicate 1
SAMEA9541426 - BDF5 Input Replicate 2
SAMEA9541427 - BDF5 Input Replicate 3

Genome browser session (e.g. UCSC)

https://tritrypdb.org/tritrypdb/jbrowse/index.html?data=%2Fa%2Fservice%2Fjbrowse%2Ftracks%2FlmexMHOMGT2001U1103&loc=LmxM.30%3A1..1427171&tracks=gene%2CBDF5_ChIP_Seq%20bdf5_elution_rep1.bw%2CBDF5_ChIP_Seq%20bdf5_elution_rep2.bw%2CBDF5_ChIP_Seq%20bdf5_elution_rep3.bw%2CBDF5_ChIP_Seq%20bdf5_input_rep_1.bw%2CBDF5_ChIP_Seq%20bdf5_input_rep_2.bw%2CBDF5_ChIP_Seq%20bdf5_input_rep_3.bw&highlight=

Methodology

Replicates

Experiment conducted in triplicate

Sequencing depth

Paired end sequencing 150 bp.

Antibodies

Pierce™ Anti-HA Magnetic Beads Catalog number: 88837 . This was verified by including a ChIP-seq against parasites that did not express the HA tagged BDF5 protein.

Peak calling parameters

Reads were quality checked and trimmed using FastQC version 11.0.5 and Cutadapt version 2.5, respectively. This was followed by alignment to the L. mexicana T7/Cas9 genome using BWA-MEM (version 0.7.17). Paired ChIP-seq and input alignment files were normalised to each other using deepTools' bamCompare (version 3.3.1) with SES normalisation and bin size of 500. Bigwig files were converted to wig files with UCSC's bigWigToWig tool, and the resulting 3 files were combined by taking the mean. Peaks were filtered to only include those with a mean log2 ratio greater than 0.5 and peaks that were less than 5 kb apart were merged. Strand switch regions were defined as regions between the end of a CDS on one strand and the beginning of CDS on the other strand. Data were visualised using IGV (Broad Institute) and Circa software (OMGenomics).

Data quality

Peaks were filtered to only include those with a mean log2 ratio greater than 0.5 and peaks that were less than 5 kb apart were merged. 175 Peaks were identified

Software

Reads were quality checked and trimmed using FastQC version 11.0.5 and Cutadapt version 2.5, respectively. This was followed by alignment to the L. mexicana T7/Cas9 genome using BWA-MEM (version 0.7.17). Paired ChIP-seq and input alignment files were normalised to each other using deepTools' bamCompare (version 3.3.1) with SES normalisation and bin size of 500. Bigwig files were converted to wig files with UCSC's bigWigToWig tool, and the resulting 3 files were combined by taking the mean. Peaks were filtered to only include those with a mean log2 ratio greater than 0.5 and peaks that were less than 5 kb apart were merged. Strand switch regions were defined as regions between the end of a CDS on one strand and the beginning of CDS on the other strand. Data were visualised using IGV (Broad Institute) and Circa software (OMGenomics).

Flow Cytometry

Plots

Confirm that:

☒ The axis labels state the marker and fluorochrome used (e.g. CD4-FITC).

☒ The axis scales are clearly visible. Include numbers along axes only for bottom left plot of group (a 'group' is an analysis of identical markers).

☒ All plots are contour plots with outliers or pseudocolor plots.

☒ A numerical value for number of cells or percentage (with statistics) is provided.

Methodology

Sample preparation

For determination of total RNA levels by SYTO RNASelect staining, 1 ml of culture was treated with 500 nM SYTO RNASelect for 20 mins at 25 oC. Cells were collected by centrifugation 1200 x g for 10 mins and washed with PBS before resuspension in PBS 10 mM EDTA pH 7.4.

Instrument

Cells were analysed using a Beckman Coulter Cyan ADP flow cytometer with detection of the stained RNA in the FL1 channel.

Software

Summit software Beckman Coulter

Cell population abundance

100% abundant - the cells are in vitro cultures of a single cell type.

Gating strategy

Cell populations were shown as histograms without gating.

☒ Tick this box to confirm that a figure exemplifying the gating strategy is provided in the Supplementary Information.
