# Supplementary material for: Bromodomain factor 5 is an essential regulator of transcription in Leishmania
Source: Nat Commun. 2022 Jul 13;13:4071. doi: 10.1038/s41467-022-31742-1 (PMC9279504; doi:10.1038/s41467-022-31742-1)

Figure 2C

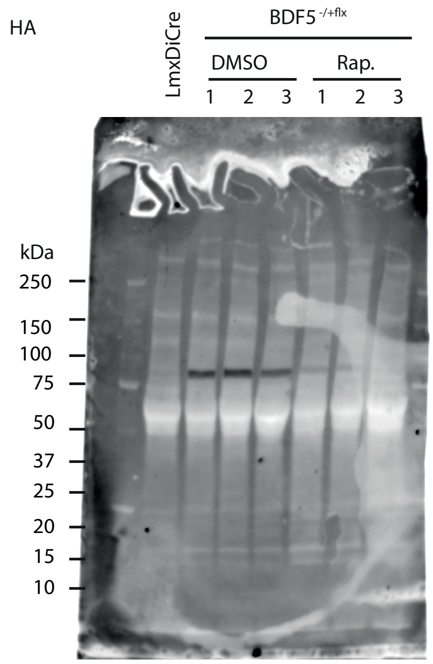

Anti-HA, Rabbit, Bethyl A190-108A
1:5000

Anti-Rabbit HRP, Goat, Promega W401B
1:5000

BioRad Clarity ECL Substrate

Expected 76.7 kDa band

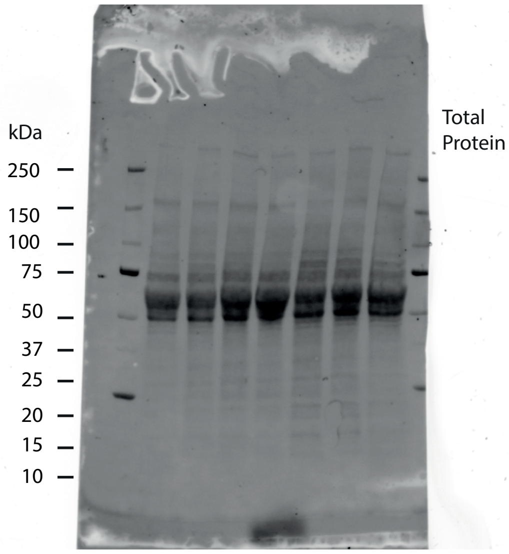

Supplementary Figure 4

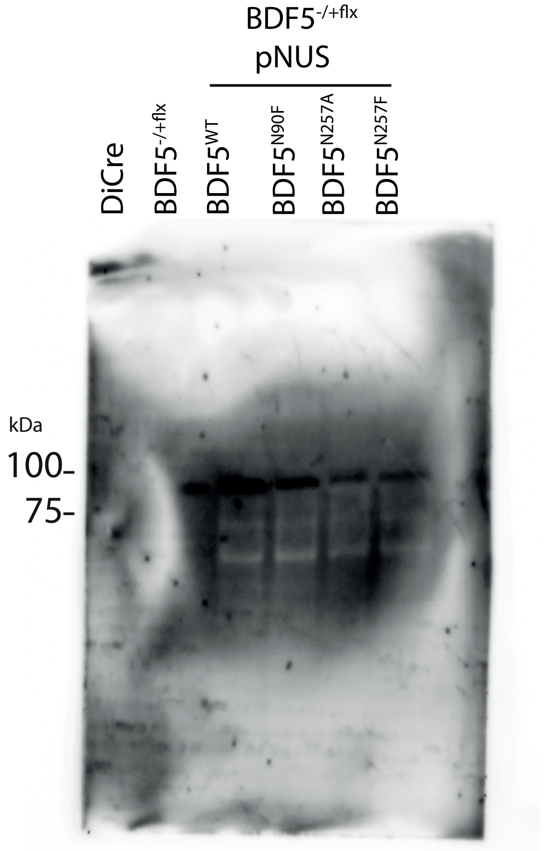

Anti-HA, Rabbit, Bethyl A190-108A
1:5000
Anti-Rabbit DyeLight 800, Goat, BioRad, SA5-10036
1:5000

Expected 76.7 kDa band

BioRad Stain Free Total Protein

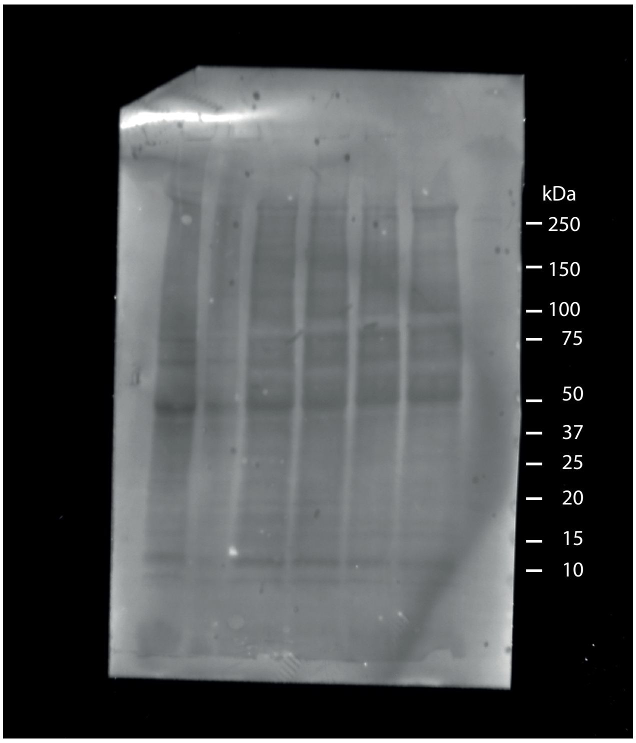

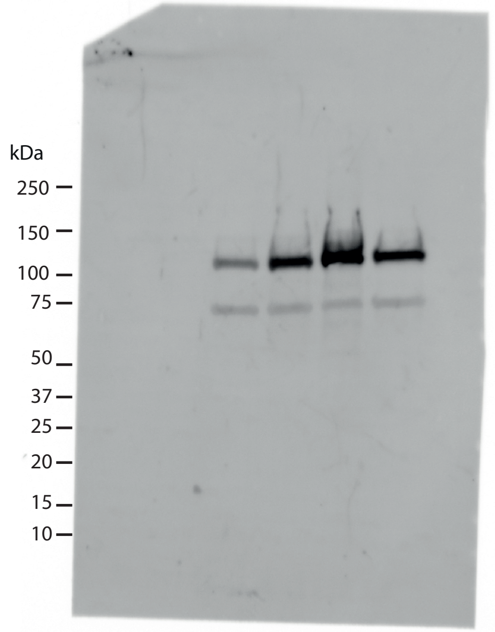

Anti-GFP JL8, Mouse, Living Colours, 632380
1:5000
Anti-mouse SB700, Goat, BioRad W401B,
1:5000

Expected 102 kDa

Supplementary Figure 5

Total Protein

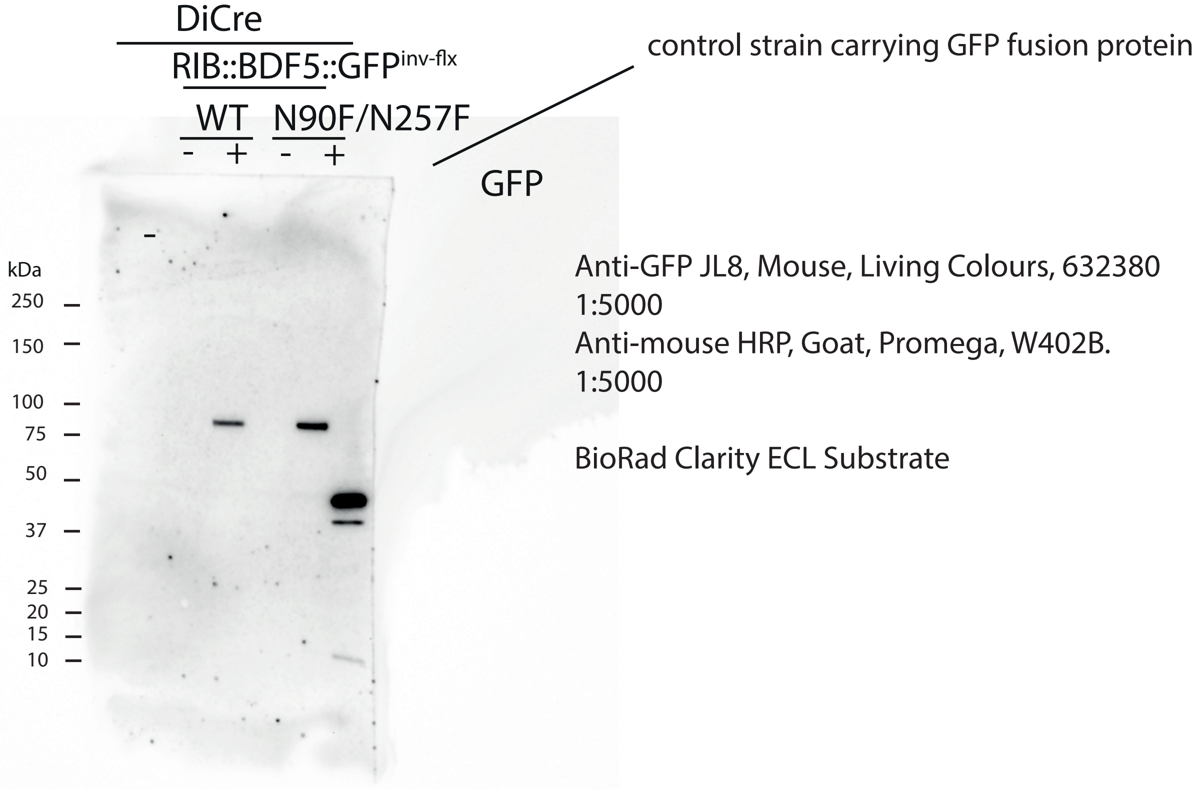

Anti-GFP JL8, Mouse, Living Colours, 632380
1:5000
Anti-mouse HRP, Goat, Promega, W402B.
1:5000

BioRad Clarity ECL Substrate

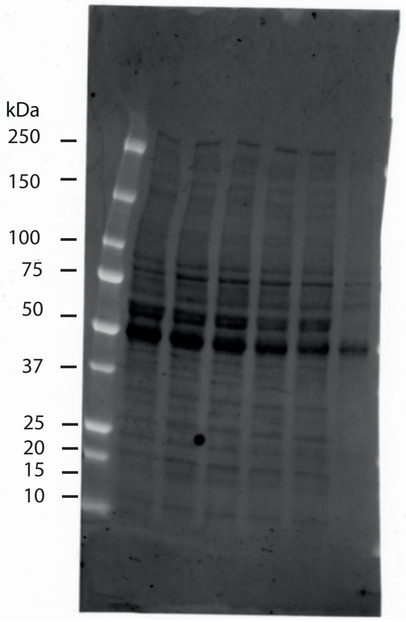

BioRad Stain-Free Total Protein

Supplementary Figure 7b,c

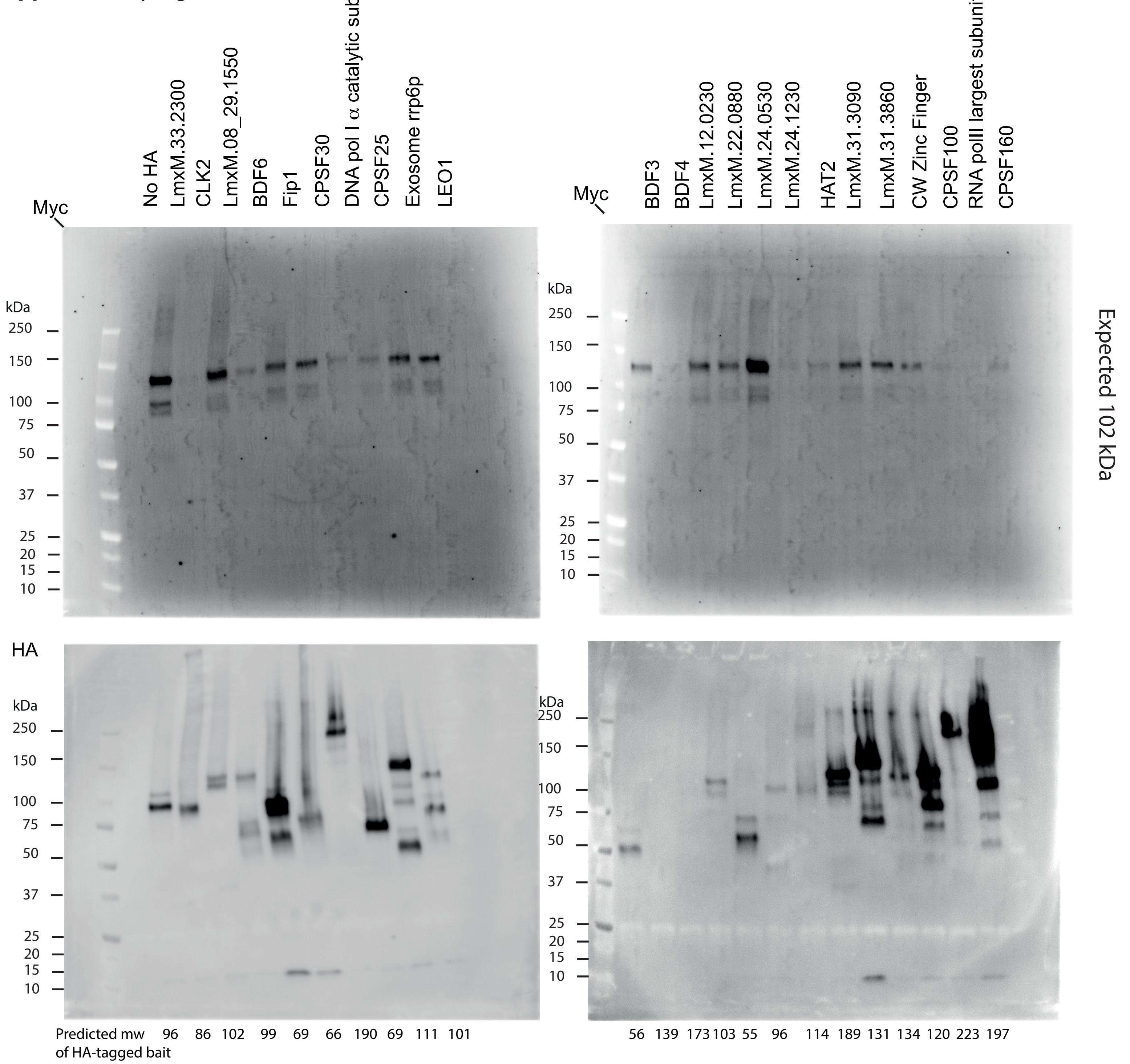

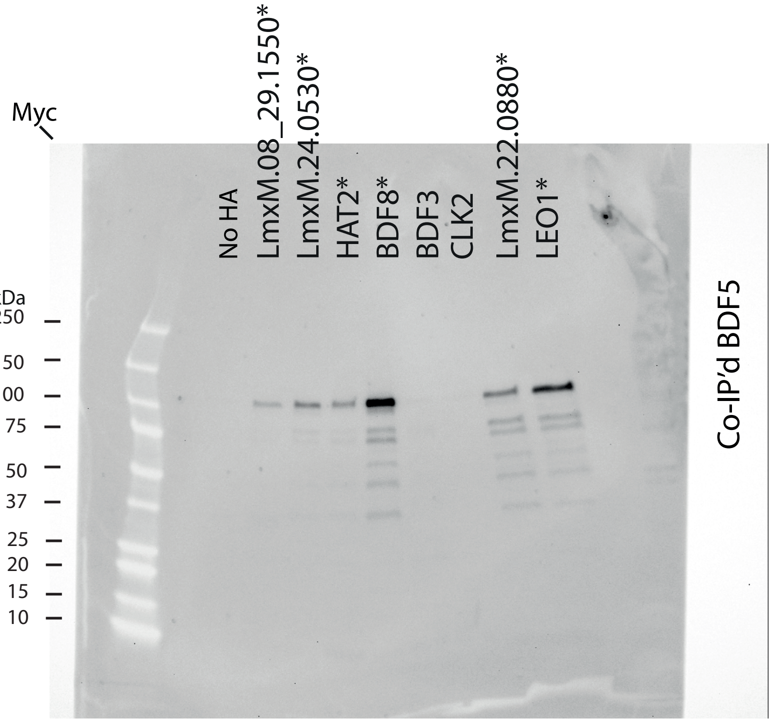

Anti-HA, Rabbit, Bethyl A190-103A
1:5000

Anti-Myc, Chicken, Bethyl A190-103A
1:5000

Anti-Rabbit SB700, Goat, BioRad, 12004162
1:5000

Anti-Chicken Dylight 800, Goat, Invitrogen, SA5-10076
1:5000

Supplementary Figure 9a

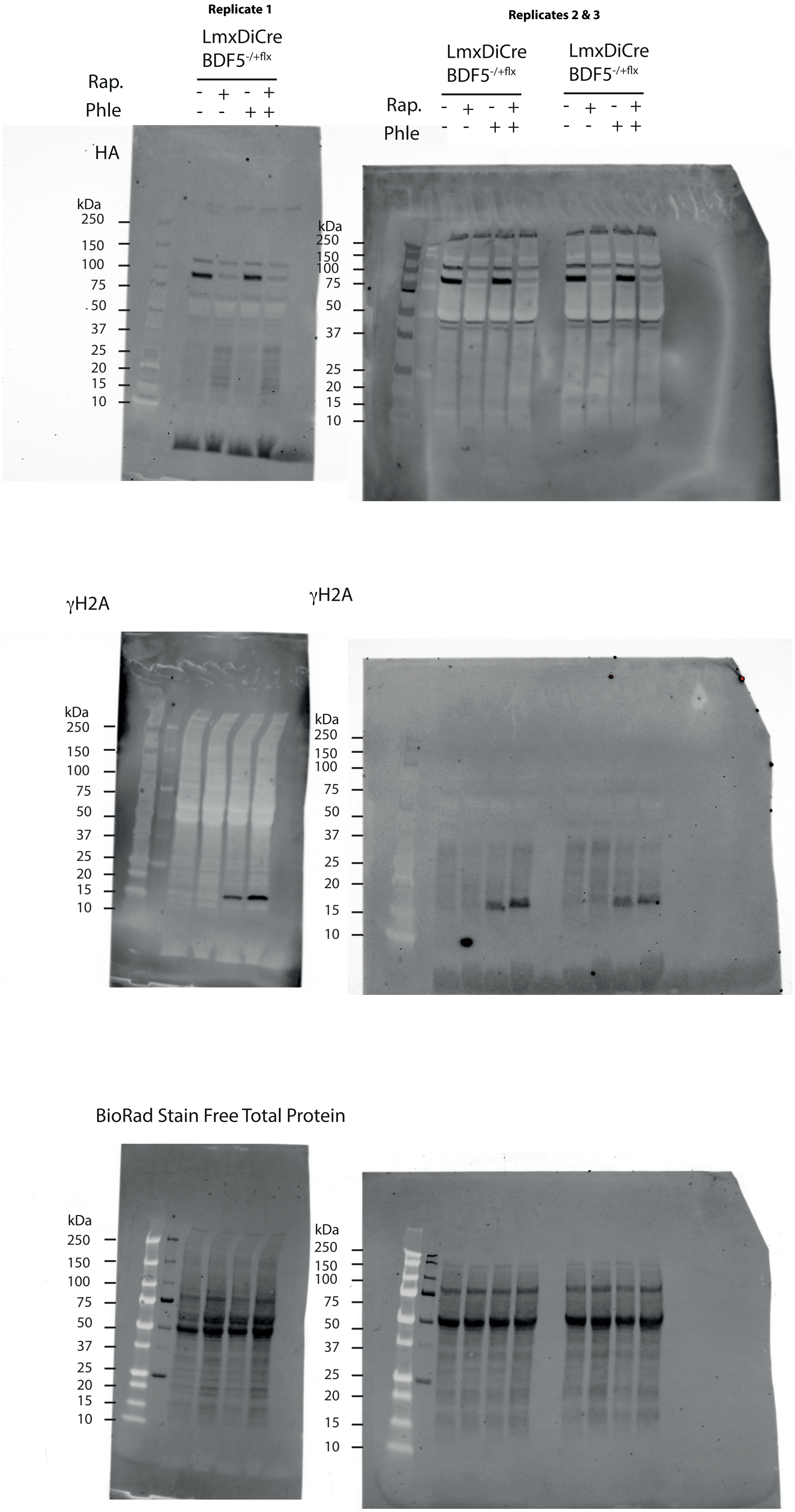

Anti-HA , Mouse 16b12,
BioLegend, 901501
1:5000
Anti-mouse SB700, Goat,
BioRad W401B,
1:5000
Expected 76.7 kDa band

Anti-gamma H2A , Rabbit,
PMID: 22353557
1:5000
Anti-Rabbit DyeLight 800, Goat,
BioRad, SA5-10036
1:5000

Expected ~20kDa band

Supplementary Figure 11b

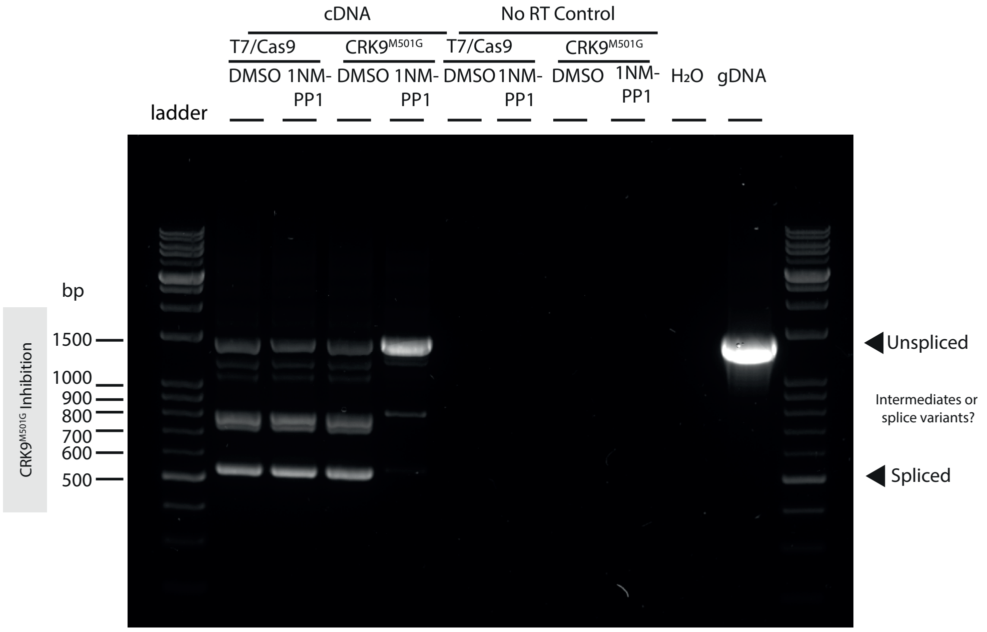

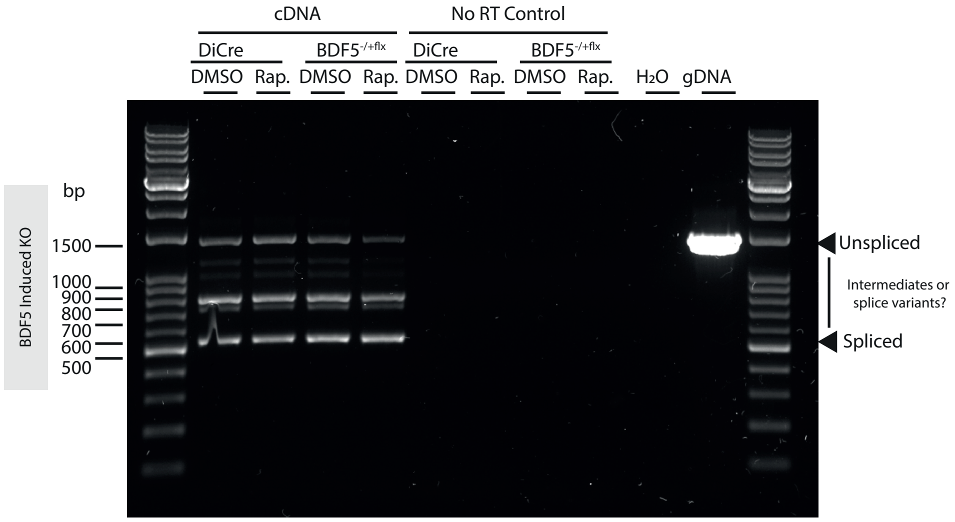

Supplementary Figure 11d

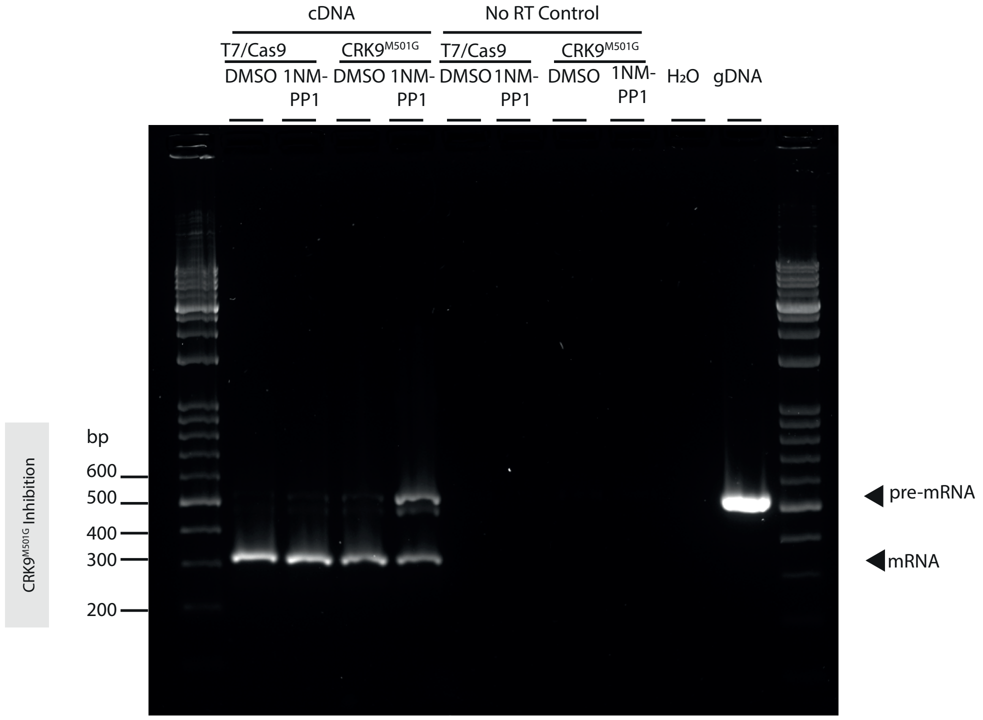

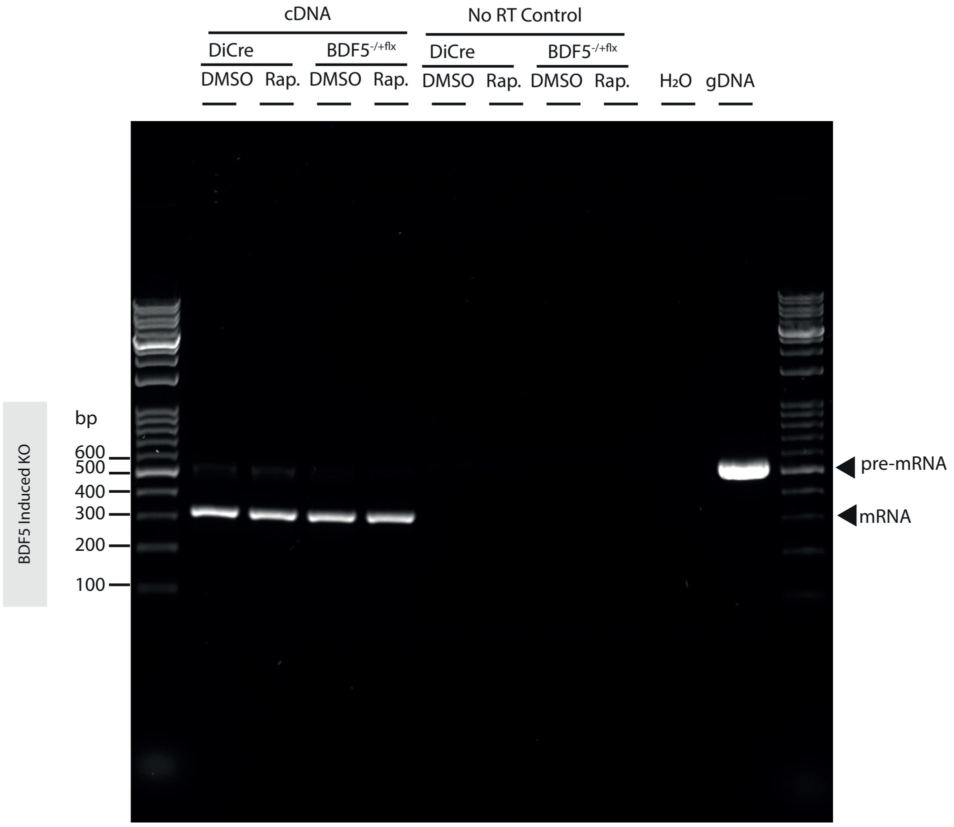

Supplement: Supplementary file 9 — Source Data [file 41467_2022_31742_MOESM9_ESM.zip › Uncropped Blots Flie BDF5 Nature Comms.pdf]
